# Optimizing the number of embryos to transfer on day 5: two should be the limit

**DOI:** 10.5935/1518-0557.20170003

**Published:** 2017

**Authors:** Claudio Ruhlmann, Lucas Molina, Graciano Tessari, Felicitas Ruhlmann, Lautaro Tessari, Diego Gnocchi, Antonio Cattaneo, Marcela Irigoyen, Alejandro Gustavo Martínez

**Affiliations:** 1 Fertilidad San Isidro, Buenos Aires, Argentina

**Keywords:** Day-5 embryo transfer, multiple pregnancies, implantation rate, blastocyst.

## Abstract

**Objective:**

To define the appropriate number of embryos to be transferred at day 5.

**Methods:**

Retrospective analysis of 784 consecutive fresh day-5 embryo transfers performed between 2007 and 2015, divided in three groups: Group A (N = 219): received the only 2 embryos that reached a transferable stage; Group B (N = 357): received 2 selected embryos among several that reached a transferable stage; Group C (N = 208): received the only 3 developing embryos. Clinical pregnancy, implantation, multiple pregnancy and delivery rates were registered. Kruskal-Wallis and Fisher Exact tests were applied as appropriate.

**Results:**

Age and previous attempts were comparable in the 3 groups. Compared with Group A, Groups B and C had a higher oocyte recovery (10.7 ± 5.6 *vs*. 14.7 ± 8.0 *vs*. 13.8 ± 6.6), fertilization rate (75.97% *vs*. 81.60% *vs*. 83.29%) and percentage of embryos reaching a transferable stage on day 5 (39.98% *vs*. 63.99% *vs*. 60.97%), as well as a significantly higher clinical pregnancy (42.92% *vs*. 61.06% *vs*. 58.17%) and implantation rates (21.09% *vs*. 40.98% *vs*. 36.97%). The multiple pregnancy rate was higher in Groups B and C than in Group A (11.70% *vs*. 31.19% *vs*. 37.19%). The high order multiple pregnancy rate (> 2) was significantly increased in group C (1.06% *vs*. 0.92% *vs*. 14.05%).

**Conclusions:**

In patients with 3 or more day 5 developing embryos, delivery rates are similar if 2 or 3 embryos are transferred. The transfer of 3 embryos carries an unacceptable increase in the risk of high order multiple pregnancy, with its known consequences. According to our data, we should not exceed the number of 2 day-5 fresh embryos transferred.

## INTRODUCTION

Multiple pregnancies have been associated to an unacceptable increase in maternal and perinatal morbidity and mortality (Crowther, 2002; Lawlo & Nelson, 2012; Luke & Brown, 2007; McDonald *et al.,* 2005). As for the fetus, a higher incidence of congenital malformations, low birth weight, prematurity, and fetal death, were reported (Dickey, 2009; Scher *et al.,* 2002). Maternal complications include an increase in the incidence of gestational hypertension, pre-eclampsia, preterm birth, premature rupture of membranes, abruptio placentae, placenta previa, gestational diabetes and cesarean section (ICMART, 2006; Dickey, 2009; Reh *et al.,* 2010).

Multiple pregnancies have also an important socio-economic impact to the health provider and the family, due to the huge increase in total costs (Campbell *et al.,* 2004; Russell *et al.,* 2007). In addition, stress generated to parents due to the emotional, economic and social impact that entails a sudden increase in the family size must be considered (Benute *et al.,* 2013; Ellison & Hall, 2003).

For all these reasons there is a global trend in favor of reducing the number of embryos to transfer (Kjellberg *et al.,* 2006; Stillman *et al.,* 2009; Straughen *et al.,* 2013), led by some European countries, where the policy of elective single embryo transfer (eSET) was adopted some time ago (Belaisch-Allart *et al.,* 2008; Pinborg *et al.,* 2003).

In our country, until the recent regulation provided by the fertility law, most treatments were paid by the patients themselves, with the consequent pressure to the medical institution to achieve a positive outcome, even at the expense of an increase in the rate of multiple pregnancies.

The objective of the present study was to compare the results obtained with the transfer of 2 or 3 embryos on day 5, in order to define the optimal number of embryos to transfer to achieve good outcomes, with an acceptable multiple pregnancy rate.

## MATERIALS AND METHODS

A retrospective analysis of 784 consecutive day 5 embryo transfers done in a private certified infertility clinic, between March 2007 and March 2014 was reported. All cycles with a fresh day 5 embryo transfer of 2 or 3 embryos in women under 40 years old using their own eggs were included. Severe male factor, advanced endometriosis, high basal FSH or previous ovarian surgery were not exclusion criteria.

All patients were stimulated under ovarian suppression with Gn-RH agonists (Lupron, Abbot Laboratories, Chicago, IL, USA), with rFSH alone Gonal-F, (Ares-Serono Laboratories, Switzerland, actually Merck Serono, Darmstadt, Germany); or Puregon, (Organon NV, Oss, The Netherlands, actually MSD, Kenilworth, NJ, USA) or combined with HMG (Menopur, Ferring Pharmaceuticals, Saint-Prex, Switzerland ), or with the same gonadotropins associated with the GnRH Antagonist Cetrorrelix 0.25 (Cetrotide 0.25, Serono Laboratories, Switzerland, actually Merck Serono, Darmstadt, Germany). An initial gonadotropin dose of 225 to 300 IU was maintained for 5 days and adjusted according to ovarian response. A single HCG dose of 10.000 IU (Gonacor 5.000, (Ferring Pharmaceuticals, Saint-Prex, Switzerland); or Pregnyl (Organon NV, The Netherlands, actually MSD, Kenilworth, NJ, USA) was administered 34-36 hours before oocyte retrieval. From the day after ovarian aspiration until pregnancy was confirmed, 800 mg of intravaginal micronized progesterone were administered daily for luteal phase support.

Four to five hours after oocyte retrieval, mature oocytes were inseminated (conventional IVF or ICSI was applied according to male evaluation) in GIVFplus medium (Vitrolife, Goteborg, Sweden). Fertilization was observed 16-18 hours after insemination. Fertilized eggs continued their development in G1 plus medium (Vitrolife, Goteborg, Sweden). On day 3, embryos were transferred to G2 plus medium (Vitrolife, Goteborg, Sweden) until day 5 or 6. The 2 or 3 more advanced developing embryos were transferred on day 5. The number of embryos transferred was defined conjointly by the treating physician, the embryologist and the couple, according to the medical history, female age, number of previous unsuccessful treatments and the embryos' developmental stage and classification. Embryo Glue (Vitrolife, Goteborg, Sweden) was used as transfer medium. Transferred embryos were classified as: compact morulae, early blastocyst, expanding blastocyst and, expanded blastocyst. In all cases, embryo transfer was done with the Frydman Ultra-soft catheter (CCD Laboratoires, Paris, France). Clinical pregnancy was initially diagnosed by serial hCG determinations and confirmed at 25-30 days after embryo transfer by transvaginal ultrasound.

The cycles were divided into 3 groups:

Group A (N = 219): patients who received the only 2 embryos that reached a transferable stage on day 5.

Group B (N = 357): patients who received 2 selected embryos among several that reached a transferable stage on day 5. Supernumerary embryos were cryopreserved.

Group C (N = 208): patients who received the only 3 developing embryos on day 5 evaluation.

The main outcome measures were: clinical pregnancy rate, implantation rate, multiple pregnancy rate and delivery rates.

Statistical comparisons were done using Kruskal-Wallis test and Fisher Exact test as appropriate, both from Instat (GraphPad Software 3.1, San Diego, CA, USA). A *p* value < 0.05 was considered significant.

### Ethical approval

The present observational comparative study was carried out in accordance with the guidelines of our Institutional Review Board (IRB). No support or economic subvention was received for the study.

### RESULTS

The 3 groups were comparable in terms of: female age, number of previous attempts and proportion of ICSI cases. Group A showed significant differences with regards to groups B and C in the number of total and mature oocytes retrieved, fertilization rate, development to the blastocyst stage, clinical pregnancy and implantation rates (Table 1).

**Table 1:** Demographic and clinical data.

	Group A Only 2 embryos	Group B 2 selected embryos	Group C Only 3 embryos
Number of embryo transfers	219	357	208
Age	35.1 ± 3.8	34.9 ± 3.9	35.0 ± 4.1
Previous attempts	2.4 ± 1.9	2.1 ± 1.2	2.5 ± 1.9
Proportion of ICSI/IVF cases	107/219 (48.86%)	180/357 (50.42%)	101/208 (48.56%)
Total oocytes retrieved	9.1 ± 4.5*	13.2 ± 6.5**	13.1 ± 6.2**
Mature oocytes retrieved	7.2 ± 3.9*	10.2 ± 5.0**	10.5 ± 4.2**
Fertilization rate	1198/1577 (75.97%)*	2971/3641 (81.60%)**	1819/2184 (83.29%)**
Blastocyst stage	479/1198 (39.98%)*	1901/2971 (63.99%)**	1109/1819 (60.97%)**

(*,**) Differ significantly (p < 0.05).

Regarding the speed of growth of the embryos on day 5, Groups A and C received a greater number of early embryos (morulae and early blastocysts) than patients in Group B, who generally received fully expanded blastocysts (Table 2).

**Table 2:** Rate of development of transferred embryos.

	Group A Only 2 embryos	Group B 2 selected embryos	Group C Only 3 embryos
Morulae	105/479 (21.92%)*	18/1901 (0.95%)**	266/1109 (23.99%)*
Early Blastocyst	144/479 (30.06%)*	95/1901 (5.00%)**	310/1109 (27.95%)*
Expanding Blastocyst	153/479 (31.94%)	514/1901 (27.04%)	322/1109 (29.04%)
Fully Expanded blastocyst	72/479 (15.03%)*	1217/1901 (64.02%)**	189/1109 (17.04%)*
Hatching Blastocyst	5/479 (1.04%)	57/1901 (3.00%)	22/1109 (1.98%)

(*,**) Differ significantly (p < 0.05).

Clinical pregnancy, embryo implantation, multiple pregnancy, and delivery rates were significantly higher in groups B and C. However, a significant increase in triplet pregnancies was evident in group C (Table 3). No neonatal death, quad or quintuplet pregnancies were recorded during the study period.

**Table 3:** Pregnancy results.

	Group A Only 2 embryos	Group B 2 selected embryos	Group C Only 3 embryos
Clinical pregnancy	94/219 (42.92%)*	218/357 (61.06%)**	121/208 (58.17%)**
Implantation	101/479 (21.09%)*	779/1901 (40.98%)**	410/1109 (36.97%)***
(≥ 2 fetuses) multiple pregnancy	11/94 (11.70%)*	68/218 (31.19%)**	45/121 (37.19%)**
High order multiple pregnancy rate	1/94 (1.06%)*	2/218 (0.92%)*	17/121 (14.05%)**
Miscarriage	11/94 (11.70%)	22/218 (10.09%)	15/121 (12.40%)
Ectopic pregnancy	1/94 (1.06%)	2/218 (0.92%)	1/121 (0.83%)
Delivery rate	82/219 (37.45%)*	194/357 (54.34%)**	105/208 (50.48%)**

(*,**,***) Differ significantly (p < 0.05).

## DISCUSSION

Multiple pregnancies are one of the major complications of ART, yielding important health risks for the babies to be born and to their mothers, and they may be minimized by adjusting the number of transferred embryos. We report our experience with day-5 embryo transfer in an unregulated scenario regarding the number of embryos to be transferred.

Patients in Group A received the only two available developing embryos on day 5. It was composed of patients with a lower ovarian response, compared to the other two groups. Through the smaller number of total and mature oocytes it was noticeable the lower rates of fertilization and development to the blastocyst stage, as well as the implantation and clinical pregnancy rates. Moreover, in this group as in Group C, the transferred embryos showed an earlier stage of development (compact morula and early blastocysts) when compared to Group B. For this reason, we can state that group A did not turn out to be as comparable as the other two groups.

Groups B (two selected embryos transferred) and Group C (only three developing embryos transferred) had, in general, a similar biologic response. Both groups were composed of patients who had 3 or more developing embryos on day 5, and exhibited similar implantation and clinical pregnancy rates. However, the rate of triplet deliveries was significantly higher in group C - 3 embryos transferred, even when the rate of development of the transferred embryos was slower, compared to the two selected embryos transferred in Group B.

At this point, we cannot ignore the advantages of a single blastocyst transfer. It has been a growing practice in recent years in many programs, as well as in ours. But due to our local conditions it's still not easy to expand its practice. The main reasons are: the couple's stress to achieve the pregnancy in the minimum time lapse, the limiting economic possibilities to afford multiple treatments, the pressure of the IVF centers to maintain high "competitive" results, the repetition of numerous treatment failures, poor embryo quality or other personal factors, leading the treating physician to increase the number of embryos to be transferred in one particular cycle ( Guidelines on number of embryos transferred, Practice Committee of the American Society for Reproductive Medicine, 2009).

According to the present outcomes, the transfer of more than two embryos on day 5 in the stimulation cycle should be avoided, due to the unacceptably high possibility of a triplet pregnancy with all its potential complications. Especially, taking into consideration that due to improvements in the embryonic culture media and the availability of very efficient vitrification methods (Mullin *et al.,* 2010), outcomes with fresh and vitrified-thawed embryos are quite comparable.

Due to the retrospective nature of the present study, results should be interpreted with caution until a prospective randomized multicenter trial is completed and confirms the present results.

## CONCLUSIONS

Since the transfer of 3 day-5 developing embryos in women under 40 years old implies a significant increase in the risk of multiple pregnancies, especially triplets, without an increase in pregnancy and implantation rates, it is advisable to limit the number of embryos to transfer on day 5 to a maximum of 2. Based on these results, regardless of the woman´s age, our IVF program has adopted a policy of limiting to a maximum of 2 the number of embryos to be transferred on day 5.
